# Explainable machine learning for predicting lower extremity deep vein thrombosis in traumatic brain injury patients: development of a SHAP-guided Random Forest model

**DOI:** 10.3389/fneur.2026.1860019

**Published:** 2026-07-28

**Authors:** Xujie Wang, Xuhui Liu, Zhiqi Yu, Kun Liu, Zihou Xing, Ming Hou, Zhan Wang

**Affiliations:** 1 Department of Emergency ICU, The Affiliated Hospital of Qinghai University, Xining, China; 2 Department of Neurology, The Second Hospital of Lanzhou University, Lanzhou, China; 3 Department of Neurology, Xinhua Hospital Affiliated with Dalian University, Dalian, China; 4 Department of Medical Engineering and Translational Applications, Qinghai University Affiliated Hospital, Xining, Qinghai, China

**Keywords:** lower extremity deep vein thrombosis, machine learning, Random Forest, SHAP, traumatic brain injury

## Abstract

**Background:**

Lower extremity deep vein thrombosis (LEDVT) is a common and clinically important complication after traumatic brain injury (TBI). Early identification of patients at increased thrombotic risk remains challenging. We aimed to develop and internally validate an interpretable machine-learning model for predicting LEDVT in patients with TBI.

**Methods:**

In this retrospective observational study, we analyzed 248 consecutive patients with TBI treated at Qinghai University Affiliated Hospital between August 2024 and December 2025. Patients were randomly assigned to a training cohort (*n* = 174) and an internal validation cohort (*n* = 74). A total of 39 demographic, clinical, and laboratory variables were screened. Feature selection was performed using least absolute shrinkage and selection operator (LASSO) regression, followed by multivariable logistic regression. Eight machine-learning algorithms were developed and compared. Model discrimination, calibration, and decision-curve performance were assessed, and the selected model was interpreted using Shapley additive explanations (SHAP).

**Results:**

LEDVT occurred in 88 of 248 patients (35.5%). LASSO retained six variables for model development: age, platelet count, D-dimer, interleukin, lower limb fracture, and prophylactic anticoagulation. In multivariable logistic regression, D-dimer, interleukin, lower limb fracture, and prophylactic anticoagulation were significantly associated with LEDVT, whereas age and platelet count showed borderline associations. In the validation cohort, the Random Forest model showed strong discrimination, with an area under the receiver operating characteristic curve of 0.931 (95% CI 0.878–0.985), sensitivity of 78.8%, specificity of 95.1%, accuracy of 87.8%, precision of 92.9%, and F1 score of 0.852. SHAP analysis identified D-dimer, interleukin, prophylactic anticoagulation, age, platelet count, and lower limb fracture as the main contributors to model output.

**Conclusion:**

An interpretable Random Forest model based on routinely available clinical and laboratory variables showed good internal predictive performance for LEDVT after TBI. After external validation, this approach may help support early risk stratification and individualized surveillance in patients at increased thrombotic risk.

## Introduction

Traumatic brain injury (TBI) is a devastating neurological disease caused by external mechanical forces, leading to structural damage or functional impairment of the brain. It is one of the leading causes of death and long-term disability worldwide. Over 50 million cases of TBI occur annually globally, and in the United States, approximately 1.7 million people seek medical attention for TBI each year, with about 275,000 being hospitalized, and 50,000 dying from this disease (1). In China, the annual incidence of TBI is similarly high, making TBI one of the leading causes of long-term disability and economic burden (2). Despite significant advances in emergency and surgical treatment, early complications, especially lower extremity deep vein thrombosis (LEDVT), remain an important factor influencing patient prognosis. TBI patients typically exhibit consciousness disturbances, memory loss, neurological deficits, and intracranial lesions confirmed by imaging studies. LEDVT, a common complication in TBI patients, can lead to more severe complications, such as pulmonary embolism (PE), which is one of the leading causes of death in TBI patients if not diagnosed and treated promptly. Garcia et al. (3) analyzed the incidence of PE in TBI patients in the United States from 2016 to 2020, finding that approximately 0.6% of TBI patients developed PE, which was associated with longer hospitalization and higher mortality. Therefore, early identification and intervention for LEDVT in TBI patients are crucial, as it not only helps reduce the occurrence of complications but also significantly improves patient survival rates and quality of life.

Although routine prophylaxis protocols, including pharmacological agents (e.g., low-molecular-weight heparin) and mechanical measures (e.g., intermittent pneumatic compression), have been widely adopted to counterbalance thromboembolic risks in neurosurgical populations, the optimal application of these strategies remains a subject of ongoing debate. As reviewed by Epstein, while appropriate prophylaxis can substantially reduce the incidence of venous thromboembolism, it also carries inherent risks, particularly intracranial hemorrhage expansion in TBI patients, necessitating a careful risk–benefit assessment (4). Ganau et al. further emphasized that prophylactic strategies must be dynamically tailored to individual patient profiles, as fixed regimens often fail to account for the heterogeneity of TBI pathophysiology (5). Moreover, comparative studies, such as the prospective work by Chibbaro et al., have demonstrated that combined approaches (pharmacological plus mechanical) may offer superior efficacy, yet their applicability in the acute phase of TBI requires further validation (6). Despite these advances in prophylaxis, the residual risk of LEDVT remains non-negligible, and current clinical practices still heavily rely on empirical judgment rather than individualized, data-driven risk stratification. This gap underscores the urgent need for a robust predictive tool that can identify high-risk patients prior to the occurrence of thromboembolic events, thereby enabling more precise and personalized prophylactic interventions.

The pathophysiology of LEDVT formation is multifactorial, involving coagulation dysfunction, inflammatory cascades, and endothelial injury. Previous studies have identified several risk factors, such as age, gender, severity of the injury, type of trauma, and whether anticoagulant therapy was administered. Among these, age is not merely a demographic variable but rather a proxy for physiological reserve and frailty, which has emerged as a critical determinant of outcomes in older adults with TBI—a population increasingly recognized as “silver trauma” patients (7). As highlighted by Depreitere et al., frailty, rather than chronological age per se, may better account for the elevated risks of complications, including thromboembolic events, in this vulnerable cohort, as it reflects the cumulative decline in multiple physiological systems that impairs the resilience to injury and subsequent stressors. This perspective suggests that the relationship between age and LEDVT risk in TBI patients may be mediated by frailty-related factors, which are often not captured by conventional risk assessments. Additionally, laboratory parameters like platelet count, interleukins, and D-dimer are also associated with deep vein thrombosis formation (8). However, the predictive ability of traditional regression models remains limited. These models often assume linear relationships, struggle to handle high-dimensional data, and fail to fully capture the complex nonlinear interactions underlying LEDVT occurrence.

In contrast, machine learning (ML) algorithms offer a promising alternative approach, capable of handling nonlinear relationships and extracting hidden patterns from complex high-dimensional datasets (9). In recent years, ML has been successfully applied in outcome prediction for various neurological and critical diseases, including spontaneous intracerebral hemorrhage, ischemic stroke, sepsis, and TBI (10–13). Compared to traditional methods, Random Forest, gradient boosting algorithms, and ensemble methods have demonstrated superior predictive performance. However, research specifically targeting TBI combined with LEDVT remains relatively limited and heterogeneous, and existing predictive methods have not been sufficiently validated for routine use. In particular, many existing methods rely on linear assumptions or limited feature integration and often provide insufficient interpretability or clinically usable risk stratification.

Given the limited and heterogeneous existing evidence, as well as the difficulty in capturing nonlinear relationships among high-dimensional clinical and laboratory predictive factors, we propose a clinical-oriented, interpretable modeling framework to conduct comprehensive evaluation (discriminative ability, calibration, and decision-analysis utility). In this context, we aim to develop and internally validate a machine learning-based predictive model for TBI combined with LEDVT and assess its application in risk stratification to support clinical management. Compared to traditional regression models, ML can better accommodate nonlinear contributions and the complex joint effects of coagulation, inflammation, and metabolic markers. We compared eight commonly used ML models under a unified feature set and evaluation framework (discriminative ability, calibration, and decision-analysis utility) and emphasized clinical translation using SHAP-based methods, providing transparent, guided decision support. Specifically, our goal is to compare the performance of different algorithms, select the optimal method, and provide an interpretable tool to transform model outputs into clinically meaningful risk stratification, bridging the gap between statistical prediction and clinical decision-making.

## Methods

**Figure 1:**
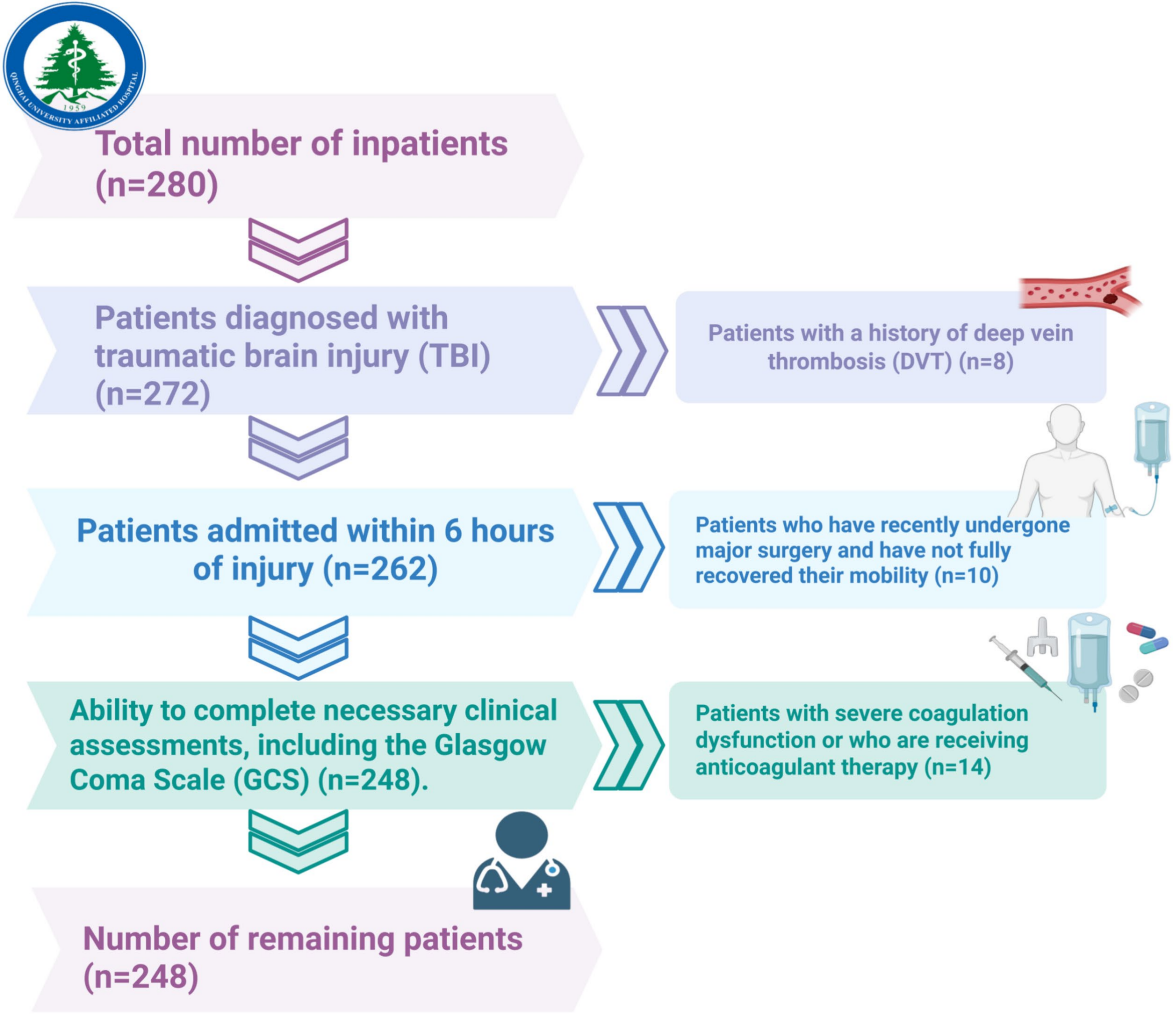
Flow diagram of the selection of eligible TBI patients.

This retrospective observational study was conducted at the Qinghai University Affiliated Hospital in China, following the RECORD (REporting of studies Conducted using Observational Routinely-collected health Data) guidelines. Clinical and laboratory data from TBI patients were extracted from the hospital’s electronic medical records system between August 2024 and December 2025. After rigorous inclusion and exclusion criteria were applied, a total of 248 patients were included. During the study period, 280 TBI patient records were screened. Among them, 8 patients with a history of deep vein thrombosis (DVT), 10 patients who had recently undergone major surgery and had not fully recovered their activity, and 14 patients with severe coagulation disorders or those undergoing anticoagulant therapy were excluded. Ultimately, 248 patients were included in the analysis. The study protocol was reviewed and approved by the Ethics Committee of Qinghai University Affiliated Hospital (approval number: SL-2026141). Given that this study was based on anonymized data and involved no identifiable patient information, informed consent was waived. All research procedures adhered to institutional and national privacy and confidentiality standards. The integrated flowchart for study screening, exclusion, and cohort assignment is shown in Figure 1.

The study subjects were patients who were admitted within 6 h of injury, diagnosed with TBI, and confirmed through cranial computed tomography (CT) or magnetic resonance imaging (MRI), and who underwent a follow-up cranial CT scan within 24 h after the initial examination. Inclusion criteria were: (1) admission within 6 h of injury; (2) confirmed diagnosis of traumatic brain injury (TBI); and (3) ability to complete necessary clinical assessments, including the Glasgow Coma Scale (GCS). The exclusion criteria were: (1) a history of DVT; (2) recent major surgery with incomplete recovery of activity; (3) severe coagulation disorders or ongoing anticoagulant therapy. A total of 248 patients were included and randomly allocated to a training cohort (*n* = 174) and a validation cohort (*n* = 74) in an approximate 7:3 ratio for model development and internal validation.

The primary outcome measure was the occurrence of TBI complicated by LEDVT, defined according to whether TBI was complicated by LEDVT: the presence of LEDVT was considered the positive criterion, and the absence of LEDVT was considered the negative criterion. A total of 39 candidate predictors were collected, including demographic factors (age, sex, smoking, hypertension, diabetes), injury-related variables (Glasgow Coma Scale, Injury Severity Score, mechanical ventilation time, lower limb immobilization, lower limb compression, lower limb fractures, brain herniation with midline shift, hospital stay duration), treatment timing and interventions (dehydration treatment and intracranial pressure control time, hypothermia treatment time, surgery timing, prophylactic anticoagulation therapy, hemostatic drugs), and laboratory parameters (white blood cell count, red blood cell count, hemoglobin, platelet count, prothrombin time, activated partial thromboplastin time, fibrinogen, fibrinogen sedimentation index, clotting time, D-dimer, triglycerides, total cholesterol, high-density lipoprotein, low-density lipoprotein, albumin, potassium, sodium, chloride, interleukin, procalcitonin).

Univariate analysis was first performed: categorical variables were analyzed using the χ^2^ test or Fisher’s exact test, and continuous variables were analyzed using the Mann–Whitney U test. Variables with *p* < 0.05 were further included in the Least Absolute Shrinkage and Selection Operator (LASSO) logistic regression, combined with 10-fold cross-validation for selection to reduce overfitting. The optimal penalty parameter (*λ* = 0.0606986) was selected based on the minimum criterion (min), and 6 predictors with non-zero coefficients were identified. These variables were then incorporated into a multivariate logistic regression model and selected using a backward stepwise regression approach. The final multivariable logistic regression model retained six variables for subsequent machine learning model development. To exclude collinearity, multicollinearity was assessed using the variance inflation factor (VIF), and no significant collinearity was found.

Based on these six retained variables (age, platelet count, D-dimer, interleukin, lower limb fracture, and prophylactic anticoagulation therapy), eight machine learning models were developed: Random Forest, GBM, XGboost, AdaBoost, SVM, Logistic, Neural Network, and KNN. The discriminative ability, calibration ability, and clinical applicability of each model were comprehensively evaluated. Discriminative ability was assessed using the area under the receiver operating characteristic curve (AUC) and metrics based on the confusion matrix, including sensitivity, specificity, precision, positive predictive value (PPV), negative predictive value (NPV), and F1 score. Calibration ability was assessed through calibration curves, the Hosmer–Lemeshow goodness-of-fit test, and Brier scores. Clinical applicability was evaluated through decision curve analysis (DCA).

Among the eight models, Random Forest demonstrated the most robust and generalized performance, maintaining high accuracy, sensitivity, and specificity in both the training and validation cohorts. To improve model interpretability, a Shapley additive explanation (SHAP) analysis was conducted on the Random Forest model to quantify the contribution of each predictor to the model’s output.

Categorical variables were presented as counts and percentages, and continuous variables were presented as medians with interquartile ranges. In intergroup comparisons, categorical variables were analyzed using the χ^2^ test or Fisher’s exact test, and continuous variables were analyzed using the Mann–Whitney U test. Logistic regression models reported odds ratios (OR) and their 95% confidence intervals (CI). All statistical analyses were performed using R software (version 4.2.1) and Python (version 3.6.5), and a two-tailed *p* < 0.05 was considered statistically significant.

## Results

A total of 248 patients with TBI were included in this study, including 190 men (76.6%) and 58 women (23.4%), with a median age of 53.0 years (IQR, 43.0–61.25 years). Of these, 174 patients were assigned to the training cohort and 74 to the validation cohort at an approximate ratio of 7:3. As shown in Table 1 and Supplementary Table S1, most baseline demographic, clinical, and laboratory characteristics were generally comparable between the training and validation cohorts; however, significant differences were observed for hypothermia timing p=0.029 and mechanical ventilation duration p=0.036 . Overall, 88 of 248 patients (35.48%) developed LEDVT. Compared with patients without LEDVT, those with LEDVT were older (median age, 56.0 vs. 50.0 years; *p* = 0.002), had lower platelet counts 159.5vs.179.0;p=0.006 , higher prothrombin time, D-dimer, and interleukin levels (all *p* < 0.001), and were more likely to have lower limb immobilization, lower limb compression, lower limb fractures, brain herniation with midline shift, and prophylactic anticoagulation therapy (all *p* ≤ 0.006). In contrast, total cholesterol was lower in the LEDVT group than in the non-LEDVT group (3.23 vs. 3.85, *p* < 0.001) (Supplementary Table S2).

**Table 1:** Baseline characteristics of the selected features in the training and validation cohorts.

Variables	Total (*n* = 248)	Test (*n* = 74)	Train (*n* = 174)	*p*	Statistic
Age, Median (Q1, Q3)	53 (43, 61.25)	55.5 (46.25, 62.75)	52 (42.25, 60)	0.151	7180.5
PLT, Median (Q1, Q3)	171 (137, 221.25)	160.5 (124.75, 216)	174 (143, 224)	0.133	5660.5
D dimer, Median (Q1, Q3)	10.9 (5, 33.08)	11.6 (6.08, 37.67)	10.65 (4.8, 25.35)	0.476	6806.5
Interleukin, Median (Q1, Q3)	28.1 (16, 72.81)	28.55 (14.9, 84.64)	28.1 (16.55, 71.66)	0.744	6,607
Lower limb fracture, *n* (%)				0.276	1.185
No	221 (89)	63 (85)	158 (91)		
Yes	27 (11)	11 (15)	16 (9)		
Prophylactic anticoagulation, *n* (%)				0.155	2.023
No	178 (72)	48 (65)	130 (75)		
Yes	70 (28)	26 (35)	44 (25)		

PLT, Platelet count.

### Variable selection by LASSO regression

**Figure 2:**
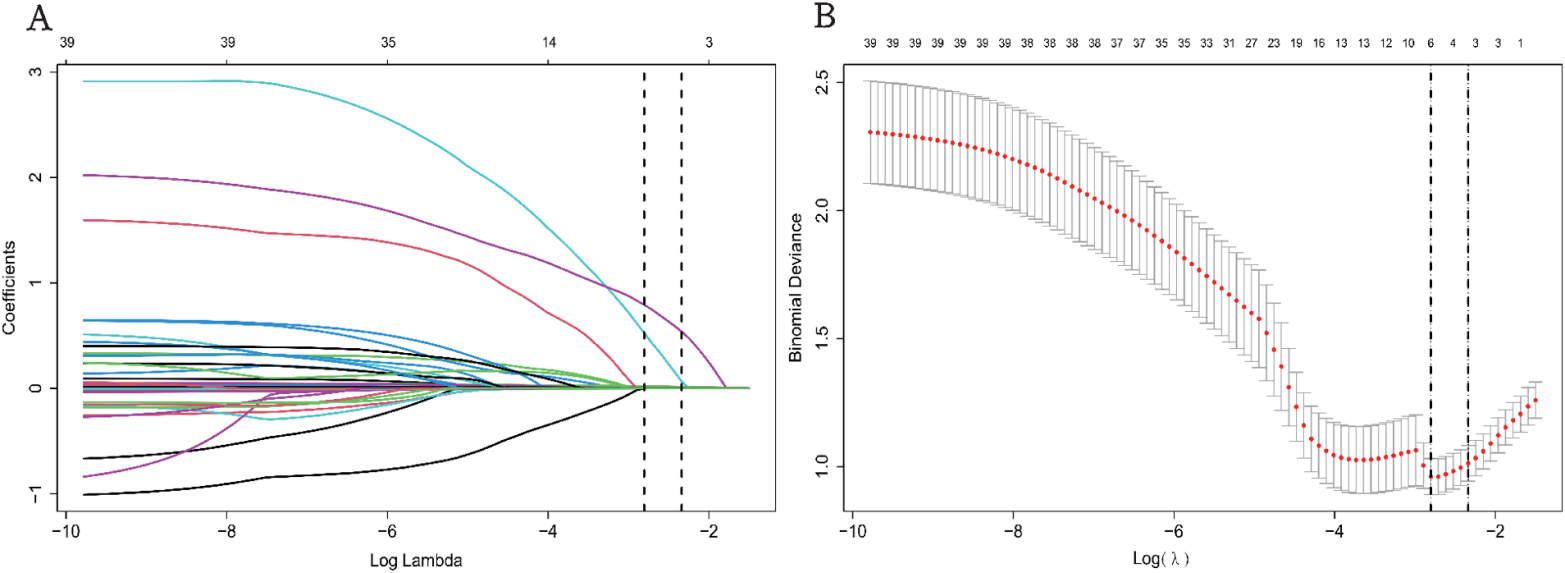
LASSO regression for feature selection. **(A)** Coefficient profiles of 39 candidate variables across log(*λ*). **(B)** Ten-fold cross-validation identifying the optimal *λ*.

To identify the most predictive variables and avoid overfitting, we employed the Least Absolute Shrinkage and Selection Operator (LASSO) logistic regression model, combined with 10-fold cross-validation for analysis. The penalty parameter *λ* was tuned using both the minimum criterion and the 1-standard error (1-SE) criterion. As shown in Figure 2A, the LASSO coefficient path plot is drawn with log(*λ*) on the x-axis; as the penalty strength increases, the coefficients of most variables gradually shrink to zero, visually demonstrating the variable selection process. Figure 2B displays the binomial deviance curve obtained through cross-validation in the main analysis cohort, where the vertical dashed lines represent the λ values corresponding to the minimum deviance (Lambda.min) and the 1-SE criterion (Lambda.1se). To enhance the model’s generalizability, we used the min criterion and selected Lambda.min = 0.0606986 as the optimal penalty parameter. The extended analysis in Supplementary Figure S1 further shows that the deviance minimization pattern was consistent across folds, confirming the stability of the model selection process. At this optimal penalty level, six predictors with non-zero coefficients were retained (see Supplementary Table S3): age (*β* = 0.001417789), plateletcount (*β* = −0.000992262), D-dimer (*β* = 0.021040576), interleukin (*β* = 0.004309478), lower limb fracture (*β* = 0.525438341), prophylactic anticoagulation therapy (*β* = 0.791964959), and the intercept (*β* = −1.754341088).

### Multivariable logistic regression model for TBI with concomitant lower extremity deep vein thrombosis

To further evaluate the variables selected by LASSO regression, we incorporated them into a multivariable logistic regression model and applied backward stepwise regression for model refinement. Ultimately, six variables were retained in the final multivariable model (Supplementary Table S4). Among them, D-dimer, interleukin, prophylactic anticoagulation, and lower limb fracture were statistically significant, whereas age and platelet count showed borderline associations. Specifically, higher D-dimer levels (OR 1.042, 95% CI 1.023–1.060; *p* < 0.001), higher interleukin levels (OR 1.013, 95% CI 1.006–1.019; *p* < 0.001), prophylactic anticoagulation therapy (OR 5.450, 95% CI 1.975–15.042; *p* = 0.001), and lower limb fracture (OR 9.760, 95% CI 1.923–49.523; *p* = 0.006) were significantly associated with LEDVT. Age showed a borderline positive association (OR 1.034, 95% CI 0.996–1.074; *p* = 0.079), whereas platelet count showed a borderline inverse association (OR 0.993, 95% CI 0.986–1.001; *p* = 0.081). The results of the variance inflation factor (VIF) analysis are shown in Supplementary Table S5, with no evidence of multicollinearity.

### Machine-learning model development and evaluation

**Figure 3:**
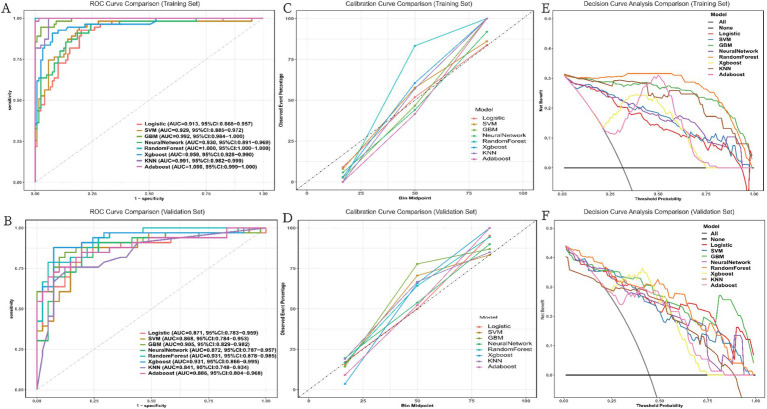
Performance of eight machine learning models in predicting concomitant lower limb deep vein thrombosis. **(A)** ROC curves in the training cohort. **(B)** ROC curves in the validation cohort. **(C)** Calibration plot in the training cohort. **(D)** Calibration plot in the validation cohort. **(E)** Decision-curve analysis (DCA) in the training cohort. **(F)** DCA in the validation cohort.

Based on these six retained variables, we built eight ML models (Random Forest, GBM, XGBoost, AdaBoost, SVM, Logistic Regression, Neural Network, and KNN) and comprehensively evaluated their performance in terms of discrimination, calibration, and clinical usefulness. To improve the model’s performance and generalizability, hyperparameter optimization was performed through grid search, with detailed results provided in Supplementary Table S6. In the validation cohort, ﻿Random Forest (AUC = 0.931, 95% CI: 0.878–0.985) demonstrated the most robust generalizability, while SVM, Logistic, and NeuralNetwork showed moderate performance (Figures 3A,B). Apart from AUC, calibration analysis indicated good consistency between the predicted probabilities and observed probabilities (Figures 3C,D); decision curve analysis showed that ﻿Random Forest continuously provided the greatest clinical net benefit across a wide range of threshold probabilities (Figures 3E,F). Other metrics, including Brier score (Supplementary Tables S7, S8) and sensitivity, specificity, precision, positive predictive value (PPV), negative predictive value (NPV), and F1 score based on the confusion matrix (Table 2; Supplementary Figure S2), as well as the direct comparison of AUC distributions across different models (Supplementary Table S9), further support this conclusion.

**Table 2:** Discrimination of eight ML models in the training and validation cohorts.

Model	Dfclass	Sensitivity	Specificity	Accuracy	Pos Pred value	Neg Pred value	Precision	F1
Logistic	dev	0.927	0.798	0.839	0.927	0.798	0.68	0.785
SVM	dev	0.945	0.807	0.851	0.945	0.807	0.693	0.800
GBM	dev	0.945	0.975	0.966	0.945	0.975	0.945	0.945
NeuralNetwork	dev	0.909	0.815	0.845	0.909	0.815	0.694	0.787
﻿Random Forest	dev	1.000	1.000	1.000	1.000	1.000	1.000	1.000
XGboost	dev	0.909	0.924	0.920	0.909	0.924	0.847	0.877
KNN	dev	1.000	0.941	0.960	1.000	0.941	0.887	0.94
AdaBoost	dev	1.000	0.983	0.989	1.000	0.983	0.965	0.982
Logistic	vad	0.788	0.854	0.824	0.788	0.854	0.812	0.800
SVM	vad	0.879	0.805	0.838	0.879	0.805	0.784	0.829
GBM	vad	0.848	0.878	0.865	0.848	0.878	0.848	0.848
NeuralNetwork	vad	0.909	0.732	0.811	0.909	0.732	0.732	0.811
Random Forest	vad	0.788	0.951	0.878	0.788	0.951	0.929	0.852
XGboost	vad	0.879	0.927	0.905	0.879	0.927	0.906	0.892
KNN	vad	0.758	0.878	0.824	0.758	0.878	0.833	0.794
AdaBoost	vad	0.758	0.902	0.838	0.758	0.902	0.862	0.806

### ﻿Random Forest as the optimal prediction model and SHAP-based explanation

Given its overall favorable and stable performance across multiple evaluation metrics, Random Forest was selected as the primary model for subsequent interpretation. ﻿Random Forest demonstrated robustness, with the optimal thresholds in the training and validation cohorts being 45.4 and 49.3%, respectively. In the validation cohort, the Random Forest model showed favorable discriminatory performance, with an AUC of 0.931 (95% CI, 0.878–0.985). According to the confusion-matrix-based metrics, the model achieved a sensitivity of 78.8%, a specificity of 95.1%, an accuracy of 87.8%, a precision of 92.9%, and an F1 score of 0.852 (Table 2; Supplementary Table S10). Radar charts displaying the performance metrics of different ML models in the training and validation cohorts (Supplementary Figures S3A,B), and heatmaps visually comparing the scores of each model on different performance metrics (Supplementary Figures S3C,D) all indicated that ﻿Random Forest performed very stably on the validation set, showing good generalization ability, further confirming its robustness compared to other algorithms.

**Figure 4:**
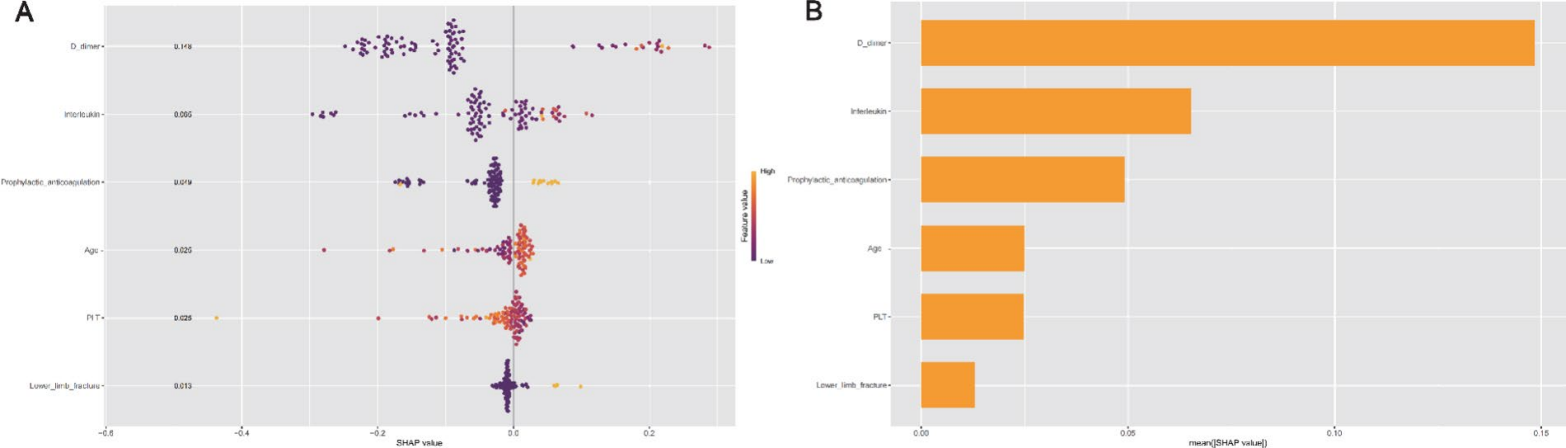
SHAP analysis of the optimal Random Forest model. **(A)** SHAP Summary plot of individual predictors. **(B)** Feature Importance ranking.

To enhance the interpretability of the ﻿Random Forest model, we introduced Shapley additive explanations (SHAP) to quantify the contribution of each predictor to the model’s output. ﻿As shown in Figure 4A,B, SHAP analysis and feature importance ranking identified D-dimer, interleukin, and prophylactic anticoagulation therapy as the most important contributors to model prediction, followed by age, platelet count, and lower limb fractures. ﻿SHAP analysis identified D-dimer, interleukin, prophylactic anticoagulation, age, platelet count, and lower limb fracture as important contributors to model output. The interpretation of effect direction should be made cautiously and in conjunction with the groupwise comparisons and multivariable logistic regression results. To provide clinically intuitive explanations beyond global feature importance, we further provided representative SHAP force/waterfall plots in Supplementary Figure S4 to illustrate how individual features contributed to model predictions at the sample level. These plots clearly demonstrate the direction and magnitude of each feature’s contribution and illustrate how key predictors collectively drive individual predictions toward higher or lower outcome risks. To further improve clinical interpretability, we also provided SHAP dependence plots for key predictors (Supplementary Figure S5) to visually identify the value ranges at which each variable begins to make a significant positive contribution to outcome prediction risk. Additionally, we plotted feature importance rankings (Supplementary Figure S6A), further emphasizing the multifactorial and interdependent nature of LEDVT, integrating multiple domains including demographics, inflammation, coagulation, and clinical factors to construct a cohesive risk framework. A multi-sample decision plot (Supplementary Figure S6B) illustrates how multiple features influence model predictions. Each line represents an instance, with color changes indicating feature value changes. This allows for a demonstration of how different features contribute to the model’s prediction and helps understand the interactions between features and their impact on final decisions. The SHAP heatmap (Supplementary Figure S6C) displays the contribution patterns of the final selected predictors in the optimal ﻿Random Forest model across different individual cases. Each row represents a predictor, and each column represents an individual sample. The color gradient from blue to pink shows the SHAP values, reflecting the positive or negative impact of each feature on the model’s output. Blue areas indicate negative contributions (lower feature values linked to lower risk), while pink areas represent positive contributions (higher feature values linked to higher risk). The SHAP main effects and interaction effect network plot (Supplementary Figure S6D) shows the relationships between features and the intensity of their interaction effects. The size of the nodes represents the importance of the features, and the lines indicate interactions between features, with colors representing the direction and intensity of influence. From the plot, the strongest interactions were found between: D-dimer and interleukin (strength: 0.0203), D-dimer and prophylactic anticoagulation therapy (strength: 0.0144), age and D-dimer (strength: 0.0099), platelet count and D-dimer (strength: 0.0081), and interleukin and prophylactic anticoagulation therapy (strength: 0.0065) (see Supplementary Table S11).

## Discussion

To date, few studies have specifically developed machine learning models for predicting LEDVT in patients with TBI. In this study, we proposed and validated an interpretable machine learning-based model to address this important clinical challenge. We constructed multiple machine learning models and used LASSO regression to select six key variables from a wide range of clinical features. Among the tested algorithms, the Random Forest model showed favorable overall performance in terms of discrimination and calibration. The main findings are summarized as follows: (1) the Random Forest model showed strong predictive performance in the validation cohort, achieving an AUC of 0.931; (2) SHapley Additive exPlanations (SHAP) identified D-dimer, interleukin, age, platelet count, lower limb fractures, and prophylactic anticoagulation therapy as the most influential predictors. Compared to previous machine learning studies on TBI, which mainly focused on broader endpoints (such as mortality or long-term functional outcomes), our strict LEDVT endpoint and multimodal predictors may partially explain the performance differences and why different algorithms perform best across studies. The tree-based ensemble framework of the Random Forest model has advantages in capturing nonlinear relationships and complex interactions between predictors, thereby overcoming some limitations of traditional regression models.

Age showed a borderline positive association with LEDVT in our cohort. Although this association did not reach conventional statistical significance in the multivariable model, it remained clinically plausible and consistent with prior evidence suggesting that older patients with TBI may be more vulnerable to thrombotic complications. Our finding that age emerged as an important predictor aligns with the concept of frailty in the silver trauma population, as recently reviewed by Depreitere et al., suggesting that physiological reserve, rather than age alone, may drive the increased thromboembolic risk in older TBI patients—a notion supported by Irimia et al.’s observation that elderly individuals are more likely to experience adverse outcomes after TBI (14). Additionally, physiological aging leads to decreased venous wall elasticity, slower blood flow, and imbalance in the hemostatic/fibrinolytic system, all of which promote thrombosis. These accumulated vulnerabilities may explain the consistent correlation between age and thrombosis.

Laboratory biomarkers provide further mechanistic insights. D-dimer is one of the strongest predictors of TBI complicated by LEDVT. Elevated D-dimer reflects active fibrinolysis and systemic coagulation activation, which are common after severe TBI, as found in Kuo et al.’s study, which demonstrated that high D-dimer levels were significantly associated with poor outcomes in TBI patients, suggesting that increased coagulation and fibrinolytic activity reflect a more complex and dynamic imbalance in hemostasis/fibrinolysis (15). Gando et al. also found that significantly elevated D-dimer levels in TBI patients were associated with a higher risk of disseminated intravascular coagulation (DIC) and secondary bleeding (16). Previous studies have shown the clinical value of D-dimer testing in venous thromboembolism (VTE) screening. For example, Akman et al. assessed the role of D-dimer in diagnosing LEDVT in rehabilitation inpatients, finding that D-dimer testing had very high sensitivity and negative predictive value (17).

Interleukin is another key predictor. LEDVT is considered an inflammatory-mediated pathological process, with inflammatory cytokines playing an important role in its occurrence and development. Studies have shown that high expression of interleukin-6 (IL-6) is closely related to the formation of LEDVT. Elevated IL-6 levels in lower extremity venous tissue and plasma may promote coagulation responses and endothelial dysfunction, thereby promoting thrombosis. Experimental evidence in mice has shown that IL-6 can enhance the formation of LEDVT by regulating inflammatory pathways (18). Additionally, large cohort studies have shown that increased levels of other interleukins, such as IL-8, are significantly correlated with the occurrence of venous thrombotic events, indicating a statistical correlation between the levels of inflammatory cytokines and thrombotic risk (19). In TBI patients, severe injury-related stress and systemic inflammatory responses are often accompanied by elevated levels of inflammatory cytokines, which may not only affect the brain injury repair process but also promote a tendency for thrombosis. Therefore, incorporating interleukin levels as a potential biomarker into the risk assessment model for TBI complicated by LEDVT is biologically plausible and helps identify high-risk individuals early and guide clinical intervention strategies.

Platelet count was also highlighted by the model as an important feature. However, its direction of association should be interpreted cautiously in the context of the present dataset. In our cohort, platelet count was lower in the LEDVT group, and in the multivariable logistic regression model it showed a borderline inverse association with the outcome. Therefore, platelet count may reflect a complex interaction between coagulation activation, platelet consumption, inflammatory status, and disease severity, rather than a simple linear increase in thrombotic risk. Platelets, as core effector cells in the hemostatic and coagulation system, reflect the coagulation status of the body through their activation levels and quantity changes. Although traditionally platelets have been associated with arterial thrombosis, increasing research indicates that platelets also play a role in various key steps in the development of venous thromboembolism (including LEDVT) (20). Platelets can promote thrombosis formation and expansion through interactions with endothelial cells, leukocytes, and von Willebrand factor; activated platelets stimulate the coagulation cascade and promote fibrin deposition, forming a stable thrombus foundation. Additionally, the cooperative effect of platelets with inflammatory cells can promote venous thrombosis development through cytokine release and the formation of neutrophil extracellular traps (NETs).

Clinical factors related to thrombosis, such as lower limb fractures and prophylactic anticoagulation therapy, are also decisive predictors. Lower limb fractures are common complications in TBI patients and are closely associated with the occurrence of LEDVT. Lower limb fractures often lead to restricted mobility, prolonged bed rest, and local trauma at the fracture site, all of which increase the risk of thrombosis by slowing blood flow. Studies have indicated that fractures significantly increase the incidence of LEDVT, suggesting that local blood flow stasis, tissue injury-induced inflammation, and prolonged immobility after lower limb fractures collectively promote the risk of venous thrombosis (21). Multicenter retrospective studies have also reported a significantly higher incidence of LEDVT in patients with lower limb fractures, particularly when there is prolonged immobilization or delayed surgery, supporting lower limb fractures as an independent risk factor for LEDVT (22).

Finally, prophylactic anticoagulation therapy deserves cautious interpretation in our model. Although prophylactic anticoagulation is generally intended to reduce the risk of venous thromboembolism, the positive association observed in the present study should not be interpreted causally. In routine clinical practice, patients judged to be at higher thrombotic risk, such as those with lower limb fracture, immobilization, or more severe overall conditions, are more likely to receive prophylactic anticoagulation. Therefore, this variable may primarily function as a marker of underlying thrombotic risk and confounding by indication, rather than evidence that prophylactic anticoagulation itself increases the risk of LEDVT. This finding should be interpreted cautiously and requires confirmation in future prospective studies.

We agree that core machine learning components (model family, internal validation strategies, and performance metrics) have been widely applied in TBI literature, and clinical translation tools such as SHAP and nomogram-style approaches have also been reported. For example, Gravesteijn et al. systematically evaluated the performance of various common machine learning algorithms (including ﻿Random Forest, SVM, etc.) and traditional regression models in predicting TBI outcomes (23). Balaji et al. used various machine learning methods (including gradient boosting trees) to predict TBI inpatient rehabilitation data and found that ML models outperformed traditional statistical models on multiple outcome metrics, identifying several important predictive features (24). Similarly, H. Khalili et al. (25) used machine learning algorithms to predict short- and long-term outcomes in TBI patients. The study showed that algorithms like ﻿Random Forest and decision trees effectively extract complex patterns from clinical data and perform better than traditional statistical methods in short-term survival prediction, providing more accurate tools for TBI prognosis assessment. It is evident that machine learning, particularly algorithms like ﻿Random Forest, SVM, and gradient boosting trees, has become an important tool for prognostic prediction. The differences between studies typically arise from outcome definitions and time windows, population severity and case mix, feature availability (especially imaging-derived variables), sample size and class imbalance, preprocessing/imputation choices, and variations in hyperparameter tuning/validation protocols. In this context, our rationale was to compare multiple established algorithms within a unified feature set and evaluation framework, selecting the most robust model for the clinically actionable endpoint of TBI complicated by LEDVT.

In conclusion, these predictive factors demonstrate the multifactorial nature of TBI complicated by LEDVT, driven by interactions between demographic variables, inflammation factors, clinical factors, and laboratory biomarkers. The fusion of vascular dysfunction, systemic inflammation, and coagulation imbalance provides a coherent mechanistic framework that explains the clinical heterogeneity of TBI and the predictive validity of our model.

In the current work, the added value lies not in the “discovery” of entirely unknown risk factors, but in quantifying and operationalizing multifactorial risks for a clinically meaningful endpoint—LEDVT, which remains highly important in clinical practice but has not been deeply explored. Specifically, (1) ML enables the integration of heterogeneous domains, including clinical factors, multi-feature characteristics, and coagulation, inflammation, and metabolic biomarkers, while capturing nonlinear contributions that are often difficult to express through linear assumptions. (2) Methodologically, we conducted rigorous feature selection and systematically compared eight ML algorithms, identifying ﻿Random Forest as the most robust model. (3) To further enhance interpretability and clinical relevance, we not only assessed discriminative ability but also evaluated calibration and decision analysis for net benefit, using SHAP to link model predictions with clinically and biologically plausible patterns.

However, several limitations should be noted. (1) As this study is based on a single-center population in China, external generalizability still needs to be validated in different ethnic and healthcare settings. (2) Despite statistical adjustments, residual confounding is inevitable in observational studies. (3) Although the model performed well in internal validation, direct head-to-head comparison with physician assessments and large-scale multicenter prospective implementation studies is needed for clinical application.

Future work is expected to further strengthen clinical translation. Although SHAP provides sample-level explanations, the current work focuses on model development and retrospective validation. Future studies will incorporate individual-level longitudinal trajectories (e.g., dynamic changes in imaging and laboratory parameters over time) to examine how changing patient conditions affect the predicted risk of LEDVT. We also plan to conduct prospective, multicenter validation and implementation studies to assess the real-world clinical impact of model-assisted decision-making. If validated externally and prospectively, the model could serve as a risk calculator at admission (e.g., integrated into electronic health records) to support early risk stratification and guide personalized decision-making for early imaging and close monitoring of high-risk patients.

These findings also carry immediate practical implications. Because the six predictors are routinely available at no additional cost, we recommend that they be documented on admission, and that patients combining elevated D-dimer, elevated interleukin, and a lower limb fracture undergo lower-extremity duplex ultrasonography at a lower threshold, with prophylaxis individualized through multidisciplinary review given the competing risk of intracranial hemorrhage expansion. In our own unit, these principles have been adopted as a preliminary quality-improvement measure—an admission checklist of the six predictors and a risk-triggered ultrasound surveillance pathway—rather than a formal change of protocol, which we consider premature until the model has been externally and prospectively validated.

## Conclusion

In this study, we developed and internally validated an interpretable Random Forest model for predicting lower extremity deep vein thrombosis in patients with traumatic brain injury. The model showed good discriminative performance and was further interpreted using SHAP, highlighting the contribution of key demographic, clinical, and laboratory variables. These findings suggest that this approach may be useful for early risk stratification in patients with TBI. Further external validation and prospective studies are needed before clinical implementation. Practically, we recommend admission-level documentation of these routinely available predictors and intensified surveillance of high-risk patients; accordingly, our unit has adopted these principles as a preliminary quality-improvement measure, reserving any formal protocol change until external and prospective validation is complete.

## Data Availability

The raw data supporting the conclusions of this article will be made available by the authors, without undue reservation.
