# Supplementary material for: Explainable machine learning for predicting lower extremity deep vein thrombosis in traumatic brain injury patients: development of a SHAP-guided Random Forest model
Source: Front Neurol. 2026 Jul 28;17:1860019. doi: 10.3389/fneur.2026.1860019 (PMC13457004; doi:10.3389/fneur.2026.1860019)
Supplement: Supplementary file 1 [file Table_1.DOCX]

***Figure S1 Cross-validation plot of LASSO regression.*** *Deviance curves across log(λ) from 10-fold cross-validation. Lambda.min was selected to balance model sparsity and performance.*

***Figure S2 Confusion matrices of 8 models in the training and validation cohorts.***
*Confusion matrices of 8 models in the training cohort (A) and validation cohort (B). Rows represent predicted classes, and columns represent reference classes. Diagonal cells indicate correct predictions; off-diagonal cells indicate errors. Numbers represent patient counts.*

***Figure S3 Performance comparison of eight machine learning models in the training and validation cohorts.***
*(A) Radar plot of each model in the training cohort (AUC, accuracy, sensitivity, specificity, precision, and F1 score).(B) Radar plot of each model in the validation cohort (AUC, accuracy, sensitivity, specificity, precision, and F1 score).(C) Heatmap of each model's performance metrics in the training cohort (F1 score, precision, specificity, sensitivity, accuracy, and AUC).(D) Heatmap of each model's performance metrics in the validation cohort (F1 score, precision, specificity, sensitivity, accuracy, and AUC).*

***Figure S4 Waterfall and Force Plots for Negative Samples.*** *(A) Waterfall plot showing individual feature contributions for negative samples.(B) Force plot illustrating the combined effect of features on predictions for negative samples.(C) Waterfall plot for a different set of negative samples.(D) Force plot for the same negative samples.*

***Figure S5 SHAP Dependence Plots for Predicting Lower Extremity Deep Vein Thrombosis in TBI Patients***
*(A) Age, (B) Interleukin, (C) D-dimer, (D) Lower limb fracture, (E) PLT, (F) Prophylactic anticoagulation.Each plot shows the relationship between the feature and its SHAP value, with the color gradient representing the feature value. The plots highlight the impact of these variables on the model’s predictions, with higher SHAP values indicating a stronger contribution to the predicted risk.*

***Figure S6 SHAP analysis visualization shows the impact of features on model output.*** *(A) The left side displays the feature importance plot, showing the contribution of each feature to the model prediction; the right side shows the SHAP summary plot, illustrating the SHAP values of each feature and their impact on the model output. (B) Multi-model decision plot, demonstrating how multiple features interact to affect the model’s predictions. (C) Heatmap, displaying the variation in feature values across different instances and their contribution to the predictions. (D) SHAP main effects and interaction effects network plot, showing the interactions between features and the strength of their influence.*

***Figure S7 ROC curves of individual predictors in the training and validation cohorts.***
*(A) ROC analysis in the training cohort.(B) ROC analysis in the validation cohort.*

***Figure S8 Decision curve analysis (DCA) of individual predictors.***
*(A) Training cohort.(B) Validation cohort.*
