# Supplementary material for: Explainable machine learning for predicting lower extremity deep vein thrombosis in traumatic brain injury patients: development of a SHAP-guided Random Forest model
Source: Front Neurol. 2026 Jul 28;17:1860019. doi: 10.3389/fneur.2026.1860019 (PMC13457004; doi:10.3389/fneur.2026.1860019)
Supplement: Supplementary file 2 [file Table_2.DOCX]

**Table S1 Baseline characteristics of the remaining variables in the training and validation cohorts.**

| **Variables** | **Total (n = 248)** | **Test (n = 74)** | **Train (n = 174)** | **p** | **Statistic** |
| --- | --- | --- | --- | --- | --- |
| Grouped, n (%) |  |  |  | 0.07 | 3.278 |
| No | 160 (65) | 41 (55) | 119 (68) |  |  |
| Yes | 88 (35) | 33 (45) | 55 (32) |  |  |
| GCS, Median (Q1,Q3) | 10 | 10 | 10 | 0.72 | 6623 |
|  | (6.75, 13) | (7, 13) | (6, 13) |  |  |
| ISS, n (%) |  |  |  | 0.597 | 0.279 |
| No | 152 (61) | 43 (58) | 109 (63) |  |  |
| Yes | 96 (39) | 31 (42) | 65 (37) |  |  |
| Dehydration ICP timing, |  |  |  | 0.451 | 1.593 |
| n (%) |  |  |  |  |  |
| No dehydration | 76 (31) | 26 (35) | 50 (29) |  |  |
| ≤24h | 129 (52) | 38 (51) | 91 (52) |  |  |
| >24h | 43 (17) | 10 (14) | 33 (19) |  |  |
| Hypothermia timing, n (%) |  |  |  | 0.029 | 7.065 |
| No dehydration | 145 (58) | 52 (70) | 93 (53) |  |  |
| ≤6h | 32 (13) | 9 (12) | 23 (13) |  |  |
| >6h | 71 (29) | 13 (18) | 58 (33) |  |  |
| Sex, n (%) |  |  |  | 0.696 | 0.153 |
| Female | 58 (23) | 19 (26) | 39 (22) |  |  |
| Man | 190 (77) | 55 (74) | 135 (78) |  |  |
| WBC, Median (Q1,Q3) | 13.84 | 12.7 | 14.39 | 0.276 | 5875 |
|  | (9.65, 17.62) | (9.04, 17.74) | (10.45, 17.46) |  |  |
| RBC, Median (Q1,Q3) | 4.74 | 4.79 | 4.72 | 0.508 | 6780.5 |
|  | (4.24, 5.31) | (4.08, 5.57) | (4.29, 5.2) |  |  |
| Hb, Median (Q1,Q3) | 147 | 151 | 146.5 | 0.209 | 7088.5 |
|  | (130, 164.25) | (132.25,173.75) | (130, 160.75) |  |  |
| PT, Median (Q1,Q3) | 11.4 | 11.4 | 11.3 | 0.763 | 6594 |
|  | (10.7, 12.1) | (10.7, 12.05) | (10.8, 12.1) |  |  |
| APTT, Median (Q1,Q3) | 24.9 | 25.15 | 24.8 | 0.341 | 6931 |
|  | (22.4, 28.1) | (23.22, 28.6) | (22.3, 27.67) |  |  |
| FIB, Median (Q1,Q3) | 2.27 | 2.36 | 2.22 | 0.989 | 6430.5 |
|  | (1.83, 2.92) | (1.83, 2.89) | (1.81, 2.96) |  |  |
| FIBS, Median (Q1,Q3) | 7.8 | 7.95 | 7.75 | 0.931 | 6483.5 |
|  | (6.2, 9.8) | (6.23, 9.7) | (6.23, 9.8) |  |  |
| TT, Median (Q1,Q3) | 17.7 | 17.85 | 17.6 | 0.479 | 6804.5 |
|  | (16.5, 19.1) | (16.83, 18.8) | (16.42, 19.1) |  |  |
| Triglycerides, | 1.06 | 1 | 1.1 | 0.525 | 6109 |
| Median (Q1,Q3) | (0.73, 1.61) | (0.75, 1.38) | (0.72, 1.69) |  |  |
| Total cholesterol, | 3.58 | 3.55 | 3.62 | 0.271 | 5868 |
| Median (Q1,Q3) | (3.1, 4.16) | (2.97, 4.12) | (3.11, 4.16) |  |  |
| HDL, Median (Q1,Q3) | 1.05 | 1.08 | 1.04 | 0.906 | 6499.5 |
|  | (0.88, 1.23) | (0.88, 1.24) | (0.89, 1.23) |  |  |
| LDL, Median (Q1,Q3) | 1.92 | 1.9 | 1.92 | 0.391 | 5994 |
|  | (1.44, 2.43) | (1.32, 2.43) | (1.51, 2.43) |  |  |
| Albumin, Median (Q1,Q3) | 37.25 | 37 | 37.45 | 0.808 | 6312 |
|  | (33.88, 40.4) | (33.82, 40.85) | (33.9, 40.05) |  |  |
| Length of stay days, | 20 | 19.5 | 20 | 0.459 | 6055 |
| Median (Q1,Q3) | (13, 32) | (13, 27) | (13.25, 33) |  |  |
| Potassium, | 3.85 | 3.84 | 3.85 | 0.858 | 6345 |
| Median (Q1,Q3) | (3.54, 4.16) | (3.51, 4.19) | (3.56, 4.15) |  |  |
| Sodium, Median (Q1,Q3) | 139 | 139.1 | 138.85 | 0.744 | 6607 |
|  | (137, 141.12) | (136.93, 141.2) | (137, 141.1) |  |  |
| Chloride, Median (Q1,Q3) | 105.1 | 105.45 | 105.1 | 0.882 | 6361 |
|  | (102.5, 107.4) | (102.17, 107.4) | (102.65, 107.4) |  |  |
| PCT, Median (Q1,Q3) | 0.21 | 0.19 | 0.21 | 0.497 | 6098 |
|  | (0.05, 0.43) | (0.05, 0.42) | (0.05, 0.44) |  |  |
| Mechanical ventilation duration, n (%) |  |  |  | 0.036 | 6.662 |
| No dehydration | 130 (52) | 47 (64) | 83 (48) |  |  |
| ≤24h | 36 (15) | 11 (15) | 25 (14) |  |  |
| >24h | 82 (33) | 16 (22) | 66 (38) |  |  |
| Smoking history, n (%) |  |  |  | 0.899 | 0.016 |
| No | 174 (70) | 51 (69) | 123 (71) |  |  |
| Yes | 74 (30) | 23 (31) | 51 (29) |  |  |
| Alcohol history, n (%) |  |  |  | 0.074 | 3.186 |
| No | 199 (80) | 65 (88) | 134 (77) |  |  |
| Yes | 49 (20) | 9 (12) | 40 (23) |  |  |
| Hypertension history, |  |  |  | 0.215 | 1.534 |
| n (%) |  |  |  |  |  |
| No | 198 (80) | 55 (74) | 143 (82) |  |  |
| Yes | 50 (20) | 19 (26) | 31 (18) |  |  |
| Diabetes history, n (%) |  |  |  | 0.433 | 0.613 |
| No | 225 (91) | 65 (88) | 160 (92) |  |  |
| Yes | 23 (9) | 9 (12) | 14 (8) |  |  |
| Lower limb immobilization, n (%) |  |  |  | 0.989 | 0 |
| No | 92 (37) | 28 (38) | 64 (37) |  |  |
| Yes | 156 (63) | 46 (62) | 110 (63) |  |  |
| Lower limb compression, |  |  |  | 0.763 | 0.091 |
| n (%) |  |  |  |  |  |
| No | 112 (45) | 35 (47) | 77 (44) |  |  |
| Yes | 136 (55) | 39 (53) | 97 (56) |  |  |
| Surgery timing, n (%) |  |  |  | 1 | Fisher |
| No dehydration | 186 (75) | 56 (76) | 130 (75) |  |  |
| ≤6h | 48 (19) | 14 (19) | 34 (20) |  |  |
| >6h | 14 (6) | 4 (5) | 10 (6) |  |  |
| Brain herniation midline shift, n (%) |  |  |  | 0.947 | 0.004 |
| No | 219 (88) | 66 (89) | 153 (88) |  |  |
| Yes | 29 (12) | 8 (11) | 21 (12) |  |  |
| Hemostatic drugs use, |  |  |  | 0.773 | 0.083 |
| n (%) |  |  |  |  |  |
| No | 200 (81) | 61 (82) | 139 (80) |  |  |
| Yes | 48 (19) | 13 (18) | 35 (20) |  |  |

Abbreviations: GCS,Glasgow Coma Scale; ISS,Injury Severity Score; ICP,Intracranial Pressure; PT,Prothrombin Time; APTT,Activated Partial Thromboplastin Time; FIB,Fibrinogen; FIBS,Fibrinogen Sedimentation Index; TT,Thrombin Time; WBC,White Blood Cell Count; RBC,Red Blood Cell Count; Hb,Hemoglobin; HDL,High-Density Lipoprotein; LDL,Low-Density Lipoprotein; PCT,Procalcitonin.

**Table S2 Comparison of baseline clinical and laboratory characteristics between patients with and without concomitant LEDVT.**

| **Variables** | **Total (n = 248)** | **No (n = 160)** | **Yes (n = 88)** | **p** | **Statistic** |
| --- | --- | --- | --- | --- | --- |
| GCS, Median (Q1,Q3) | 10 (6.75, 13) | 10 (7, 14) | 10 (6, 12) | 0.096 | 7935.5 |
| ISS, n (%) |  |  |  | 0.08 | 3.075 |
| No | 152 (61) | 105 (66) | 47 (53) |  |  |
| Yes | 96 (39) | 55 (34) | 41 (47) |  |  |
| Dehydration ICP timing, n (%) |  |  |  | < 0.001 | 14.541 |
| No dehydration | 76 (31) | 55 (34) | 21 (24) |  |  |
| ≤24h | 129 (52) | 88 (55) | 41 (47) |  |  |
| >24h | 43 (17) | 17 (11) | 26 (30) |  |  |
| Hypothermia timing, n (%) |  |  |  | < 0.001 | 14.071 |
| No dehydration | 145 (58) | 106 (66) | 39 (44) |  |  |
| ≤6h | 32 (13) | 13 (8) | 19 (22) |  |  |
| >6h | 71 (29) | 41 (26) | 30 (34) |  |  |
| Sex, n (%) |  |  |  | 0.773 | 0.083 |
| Female | 58 (23) | 36 (22) | 22 (25) |  |  |
| Man | 190 (77) | 124 (78) | 66 (75) |  |  |
| Age, Median (Q1,Q3) | 53  (43, 61.25) | 50  (39.75, 60) | 56  (48.75, 62.25) | 0.002 | 5349.5 |
| WBC, Median (Q1,Q3) | 13.84  (9.65, 17.62) | 13.32  (9.33, 16.63) | 14.86  (11.21, 18.6) | 0.03 | 5869.5 |
| RBC, Median (Q1,Q3) | 4.74  (4.24, 5.31) | 4.77  (4.32, 5.33) | 4.64  (3.88, 5.23) | 0.142 | 7834 |
| Hb, Median (Q1,Q3) | 147  (130, 164.25) | 147  (131, 161) | 148.5  (127.75, 168) | 0.891 | 6965.5 |
| PLT, Median (Q1,Q3) | 171  (137, 221.25) | 179  (145.75, 226) | 159.5  (126.25, 197.25) | 0.006 | 8530.5 |
| PT, Median (Q1,Q3) | 11.4  (10.7, 12.1) | 11.1  (10.67, 11.8) | 11.6  (11.1, 12.53) | < 0.001 | 4910.5 |
| APTT, Median (Q1,Q3) | 24.9  (22.4, 28.1) | 25.4  (22.87, 28.63) | 24.05  (22.12, 27.07) | 0.054 | 8081 |
| FIB, Median (Q1,Q3) | 2.27  (1.83, 2.92) | 2.29  (1.84, 2.92) | 2.24  (1.74, 2.87) | 0.616 | 7311.5 |
| FIBS, Median (Q1,Q3) | 7.8  (6.2, 9.8) | 7.5  (6.1, 9.53) | 8.4  (7.07, 10.7) | 0.039 | 5922.5 |
| TT, Median (Q1,Q3) | 17.7  (16.5, 19.1) | 17.75  (16.78, 18.8) | 17.55  (16.17, 19.35) | 0.817 | 7165.5 |
| D dimer, Median (Q1,Q3) | 10.9  (5, 33.08) | 7.3  (3.2, 11.15) | 36.35  (17.32, 61.28) | < 0.001 | 1826.5 |
| Triglycerides, Median (Q1,Q3) | 1.06  (0.73, 1.61) | 1.07  (0.76, 1.74) | 1.02  (0.65, 1.38) | 0.041 | 8145.5 |
| Total cholesterol,  Median (Q1,Q3) | 3.58  (3.1, 4.16) | 3.85  (3.21, 4.3) | 3.23  (2.94, 3.66) | < 0.001 | 9386 |
| HDL, Median (Q1,Q3) | 1.05  (0.88, 1.23) | 1.05  (0.94, 1.2) | 1.06  (0.74, 1.26) | 0.626 | 7304 |
| LDL, Median (Q1,Q3) | 1.92  (1.44, 2.43) | 1.96  (1.56, 2.5) | 1.79  (1.35, 2.28) | 0.015 | 8361 |
| Albumin, Median (Q1,Q3) | 37.25  (33.88, 40.4) | 37.45  (34.2, 40.68) | 36.75  (32.77, 39.73) | 0.121 | 7879 |
| Length of stay days,  Median (Q1,Q3) | 20  (13, 32) | 19  (12.75, 28.25) | 22.5  (14, 36) | 0.081 | 6097 |
| Potassium, Median (Q1,Q3) | 3.85  (3.54, 4.16) | 3.9  (3.6, 4.16) | 3.7  (3.4, 4.16) | 0.012 | 8399 |
| Sodium, Median (Q1,Q3) | 139  (137, 141.12) | 139.15  (137.1, 141.2) | 138.7  (136.93, 141) | 0.621 | 7307.5 |
| Chloride, Median (Q1,Q3) | 105.1  (102.5, 107.4) | 105.1  (102.95, 107.32) | 105.05  (101.95, 108) | 0.999 | 7038.5 |
| Interleukin, Median (Q1,Q3) | 28.1  (16, 72.81) | 22.6  (14.4, 30.1) | 74.22  (28.1, 155.48) | < 0.001 | 3263 |
| PCT, Median (Q1,Q3) | 0.21  (0.05, 0.43) | 0.22  (0.05, 0.42) | 0.11  (0.05, 0.45) | 0.358 | 7521 |
| Mechanical ventilation duration, n (%) |  |  |  | 0.154 | 3.746 |
| No dehydration | 130 (52) | 91 (57) | 39 (44) |  |  |
| ≤24h | 36 (15) | 22 (14) | 14 (16) |  |  |
| >24h | 82 (33) | 47 (29) | 35 (40) |  |  |
| Smoking history, n (%) |  |  |  | 0.516 | 0.423 |
| No | 174 (70) | 115 (72) | 59 (67) |  |  |
| Yes | 74 (30) | 45 (28) | 29 (33) |  |  |
| Alcohol history, n (%) |  |  |  | 0.103 | 2.653 |
| No | 199 (80) | 123 (77) | 76 (86) |  |  |
| Yes | 49 (20) | 37 (23) | 12 (14) |  |  |
| Hypertension history, n (%) |  |  |  | 0.362 | 0.832 |
| No | 198 (80) | 131 (82) | 67 (76) |  |  |
| Yes | 50 (20) | 29 (18) | 21 (24) |  |  |
| Diabetes history, n (%) |  |  |  | 0.094 | 2.806 |
| No | 225 (91) | 141 (88) | 84 (95) |  |  |
| Yes | 23 (9) | 19 (12) | 4 (5) |  |  |
| Lower limb immobilization,  n (%) |  |  |  | < 0.001 | 17.314 |
| No | 92 (37) | 75 (47) | 17 (19) |  |  |
| Yes | 156 (63) | 85 (53) | 71 (81) |  |  |
| Lower limb compression,  n (%) |  |  |  | 0.006 | 7.46 |
| No | 112 (45) | 83 (52) | 29 (33) |  |  |
| Yes | 136 (55) | 77 (48) | 59 (67) |  |  |
| Lower limb fracture, n (%) |  |  |  | < 0.001 | 21.647 |
| No | 221 (89) | 154 (96) | 67 (76) |  |  |
| Yes | 27 (11) | 6 (4) | 21 (24) |  |  |
| Prophylactic anticoagulation,  n (%) |  |  |  | < 0.001 | 30.277 |
| No | 178 (72) | 134 (84) | 44 (50) |  |  |
| Yes | 70 (28) | 26 (16) | 44 (50) |  |  |
| Surgery timing, n (%) |  |  |  | 0.091 | Fisher |
| No dehydration | 186 (75) | 127 (79) | 59 (67) |  |  |
| ≤6h | 48 (19) | 25 (16) | 23 (26) |  |  |
| >6h | 14 (6) | 8 (5) | 6 (7) |  |  |
| Brain herniation midline shift, n (%) |  |  |  | 0.003 | 8.866 |
| No | 219 (88) | 149 (93) | 70 (80) |  |  |
| Yes | 29 (12) | 11 (7) | 18 (20) |  |  |
| Hemostatic drugs use, n (%) |  |  |  | 0.607 | 0.265 |
| No | 200 (81) | 127 (79) | 73 (83) |  |  |
| Yes | 48 (19) | 33 (21) | 15 (17) |  |  |

Abbreviations:Abbreviations: GCS,Glasgow Coma Scale; ISS,Injury Severity Score; ICP,Intracranial Pressure; PT,Prothrombin Time; APTT,Activated Partial Thromboplastin Time; FIB,Fibrinogen; FIBS,Fibrinogen Sedimentation Index; TT,Thrombin Time; WBC,White Blood Cell Count; RBC,Red Blood Cell Count; Hb,Hemoglobin; PLT,Platelet Count; HDL,High-Density Lipoprotein; LDL,Low-Density Lipoprotein; PCT,Procalcitonin.

**Table S3 Variables selected by LASSO regression with corresponding coefficients at Lambda.min.**

| **Variable** | **Coefficient (β)** |
| --- | --- |
| Intercept | -1.754341088 |
| Age | 0.001417789 |
| PLT | -0.000992262 |
| D dimer | 0.021040576 |
| Interleukin | 0.004309478 |
| Lower limb fracture | 0.525438341 |
| Prophylactic anticoagulation | 0.791964959 |

**Table S4 Multivariate Logistic Regression Analysis of Independent Predictors for LEDVT in TBI Patients.**

| **Characteristics** | **B** | **SE** | **OR** | **CI** | **Z** | **P** | **P_raw** |
| --- | --- | --- | --- | --- | --- | --- | --- |
| Intercept | -3.894 | 1.4224 | 0.02 | 0.020 (0.001–0.331) | -2.738 | <0.01 | 0.006 |
| D dimer | 0.041 | 0.00904 | 1.042 | 1.042 (1.023–1.060) | 4.509 | <0.001 | 0 |
| Interleukin | 0.013 | 0.0033 | 1.013 | 1.013 (1.006–1.019) | 3.789 | <0.001 | 0 |
| Prophylactic anticoagulation | 1.696 | 0.51797 | 5.450 | 5.450 (1.975–15.042) | 3.274 | <0.01 | 0.001 |
| Lower limb fracture | 2.278 | 0.82867 | 9.760 | 9.760 (1.923–49.523) | 2.749 | <0.01 | 0.006 |
| Age | 0.034 | 0.01926 | 1.034 | 1.034 (0.996–1.074) | 1.757 | 0.079 | 0.079 |
| PLT | -0.007 | 0.00382 | 0.993 | 0.993 (0.986–1.001) | -1.748 | 0.081 | 0.081 |

**Table S5 Variance inflation factor (VIF) values for variables included in the multivariate logistic regression model.**

|  | **VIF** |  |
| --- | --- | --- |
| Age | 1.084753574 | |
| PLT | 1.074155371 | |
| D dimer | 1.039052412 | |
| Interleukin | 1.108266631 | |
| Lower limb fracture | 1.073760341 | |
| Prophylactic anticoagulation | 1.071085784 | |

**Table S6 Hyperparameters optimized for each machine learning model through grid search.**

| **Model** | **Tuning Method** | **Best Hyperparameters** |
| --- | --- | --- |
| RandomForest | Grid search | mtry = 2 |
| GBM | Grid search | n.trees = 100, interaction.depth = 2, shrinkage = 0.1, n.minobsinnode = 5 |
| Xgboost | Grid search | nrounds = 10, max_depth = 3, eta = 0.1, gamma = 0.5, colsample_bytree = 0.5, min_child_weight = 1, subsample = 0.6 |
| Adaboost | Grid search | mfinal = 30, maxdepth = 2, coeflearn = 1 |
| SVM | Grid search | sigma = 0.1, C = 0.5 |
| Logistic | Grid search | parameter = none |
| NeuralNetwork | Grid search | size = 5, decay = 0.6 |
| KNN | Grid search | kmax = 15, distance = 1, kernel = 1 |

**Table S7 Brier scores of eight ML models in the training cohort.**

| **Name** | **bs_score** |
| --- | --- |
| RandomForest | brier_score=0.0148(0.0110-0.0188) |
| XGBoost | brier_score=0.0121(0.0084-0.0165) |
| GradientBoosting | brier_score=0.0039(0.0024-0.0057) |
| AdaBoost | brier_score=0.1491(0.1406-0.1579) |
| LogisticReg_LinearReg | brier_score=0.1150(0.0872-0.1448) |
| ElasticNetCV | brier_score=0.1497(0.1195-0.1832) |
| SVM | brier_score=0.1463(0.1173-0.1779) |
| KNN | brier_score=0.1297(0.1024-0.1588) |

**Table S8 Brier scores of eight ML models in the validation cohort.**

| **Name** | **bs_score** |
| --- | --- |
| RandomForest | brier_score=0.1153(0.0699-0.1644) |
| XGBoost | brier_score=0.1028(0.0533-0.1574) |
| GradientBoosting | brier_score=0.1394(0.0821-0.2055) |
| AdaBoost | brier_score=0.1662(0.1432-0.1900) |
| LogisticReg_LinearReg | brier_score=0.1359(0.0828-0.1944) |
| ElasticNetCV | brier_score=0.1596(0.1105-0.2154) |
| SVM | brier_score=0.1480(0.1012-0.2003) |
| KNN | brier_score=0.1472(0.0955-0.2053) |

**Table S9 Area under the ROC curve (AUC) with 95% confidence intervals for eight ML models in the training and validation cohorts.**

| **Model** | **Dfclass** | **AUC** | **Low** | **Up** |
| --- | --- | --- | --- | --- |
| Logistic | dev | 0.913 | 0.868 | 0.957 |
| SVM | dev | 0.929 | 0.885 | 0.972 |
| GBM | dev | 0.992 | 0.984 | 1.000 |
| NeuralNetwork | dev | 0.930 | 0.891 | 0.969 |
| RandomForest | dev | 1.000 | 1.000 | 1.000 |
| Xgboost | dev | 0.959 | 0.928 | 0.990 |
| KNN | dev | 0.991 | 0.982 | 0.999 |
| Adaboost | dev | 1.000 | 0.999 | 1.000 |
| Logistic | vad | 0.871 | 0.783 | 0.959 |
| SVM | vad | 0.868 | 0.784 | 0.953 |
| GBM | vad | 0.905 | 0.829 | 0.982 |
| NeuralNetwork | vad | 0.872 | 0.787 | 0.957 |
| RandomForest | vad | 0.931 | 0.878 | 0.985 |
| Xgboost | vad | 0.931 | 0.866 | 0.995 |
| KNN | vad | 0.841 | 0.748 | 0.934 |
| Adaboost | vad | 0.886 | 0.804 | 0.968 |

**Table S10 Evaluation of the Random Forest model's classification performance on different datasets (raw data and binary data). The table presents various evaluation metrics, including Accuracy (ACC), Area Under the Curve (AUC), Sensitivity (SEN), Specificity (SPE), Positive Predictive Value (PPV), and Negative Predictive Value (NPV), among others.**

| **Datatset** | **Trianset raw** | **Trianset binary** | **Validationset raw** | **Validationset binary** |
| --- | --- | --- | --- | --- |
| **Cutoff** | 0.226 | 0.226 | 0.358 | 0.226 |
| **AUC** | 0.913 | 0.863 | 0.871 | 0.802 |
| **AUC.SE** | 0.023 | 0.036 | 0.045 | 0.065 |
| **AUC.low** | 0.868 | 0.792 | 0.783 | 0.675 |
| **AUC.up** | 0.957 | 0.933 | 0.959 | 0.929 |
| **ACC** | 0.839 | 0.839 | 0.824 | 0.797 |
| **ACC.low** | 0.838 | 0.838 | 0.82 | 0.793 |
| **ACC.up** | 0.841 | 0.841 | 0.828 | 0.802 |
| **SEN** | 0.927 | 0.927 | 0.788 | 0.848 |
| **SEN.low** | 0.859 | 0.859 | 0.648 | 0.726 |
| **SEN.up** | 0.996 | 0.996 | 0.927 | 0.971 |
| **SPE** | 0.798 | 0.798 | 0.854 | 0.756 |
| **SPE.low** | 0.726 | 0.726 | 0.745 | 0.625 |
| **SPE.up** | 0.87 | 0.87 | 0.962 | 0.888 |
| **PLR** | 4.598 | 4.598 | 5.384 | 3.479 |
| **PLR.low** | 3.192 | 3.192 | 2.517 | 1.991 |
| **PLR.up** | 6.623 | 6.623 | 11.514 | 6.077 |
| **NLR** | 0.091 | 0.091 | 0.248 | 0.2 |
| **NLR.low** | 0.035 | 0.035 | 0.127 | 0.088 |
| **NLR.up** | 0.235 | 0.235 | 0.485 | 0.458 |
| **PPV** | 0.68 | 0.68 | 0.812 | 0.737 |
| **PPV.low** | 0.574 | 0.574 | 0.677 | 0.597 |
| **PPV.up** | 0.786 | 0.786 | 0.948 | 0.877 |
| **NPV** | 0.96 | 0.96 | 0.833 | 0.861 |
| **NPV.low** | 0.921 | 0.921 | 0.721 | 0.748 |
| **NPV.up** | 0.998 | 0.998 | 0.946 | 0.974 |
| **PPA** | 0.927 | 0.927 | 0.788 | 0.848 |
| **PPA.low** | 0.859 | 0.859 | 0.648 | 0.726 |
| **PPA.up** | 0.996 | 0.996 | 0.927 | 0.971 |
| **NPA** | 0.798 | 0.798 | 0.854 | 0.756 |
| **NPA.low** | 0.726 | 0.726 | 0.745 | 0.625 |
| **NPA.up** | 0.87 | 0.87 | 0.962 | 0.888 |
| **TPA** | 0.839 | 0.839 | 0.824 | 0.797 |
| **TPA.low** | 0.784 | 0.784 | 0.738 | 0.706 |
| **TPA.up** | 0.894 | 0.894 | 0.911 | 0.889 |
| **KAPPA** | 0.661 | 0.661 | 0.643 | 0.596 |
| **KAPPA.low** | 0.55 | 0.55 | 0.468 | 0.415 |
| **KAPPA.up** | 0.772 | 0.772 | 0.819 | 0.777 |

Abbreviations: ACC,Accuracy; AUC,Area Under the Curve; SEN,Sensitivity; SPE,Specificity; PPV,Positive Predictive Value; NPV, Negative Predictive Value; PPA,Positive Percent Agreement; NPA, Negative Percent Agreement; TPA,Total Percent Agreement; Kappa,Kappa Statistic.

**Table S11 The table of the top five strongest feature interactions.**

|  | **Strength** |
| --- | --- |
| D-dimer → Interleukin | 0.0203 |
| D-dimer → Prophylactic anticoagulation therapy | 0.0144 |
| Age → D-dimer | 0.0099 |
| Platelet count → D-dimer | 0.0081 |
| Interleukin → Prophylactic anticoagulation therapy | 0.0065 |
